# Supplementary material for: Locomotor Strategy to Perform 6-Minute Walk Test in People with Multiple Sclerosis: A Prospective Observational Study
Source: Sensors (Basel). 2023 Mar 24;23(7):3407. doi: 10.3390/s23073407 (PMC10099238; doi:10.3390/s23073407)
Supplement: Supplementary file 1 [file sensors-23-03407-s001.zip › sensors-2069739-supplementary.pdf]

Supplementary data SA: Spatio-temporal variables during each interval of the 6MWT for PwMS with moderate and severe MS.

1

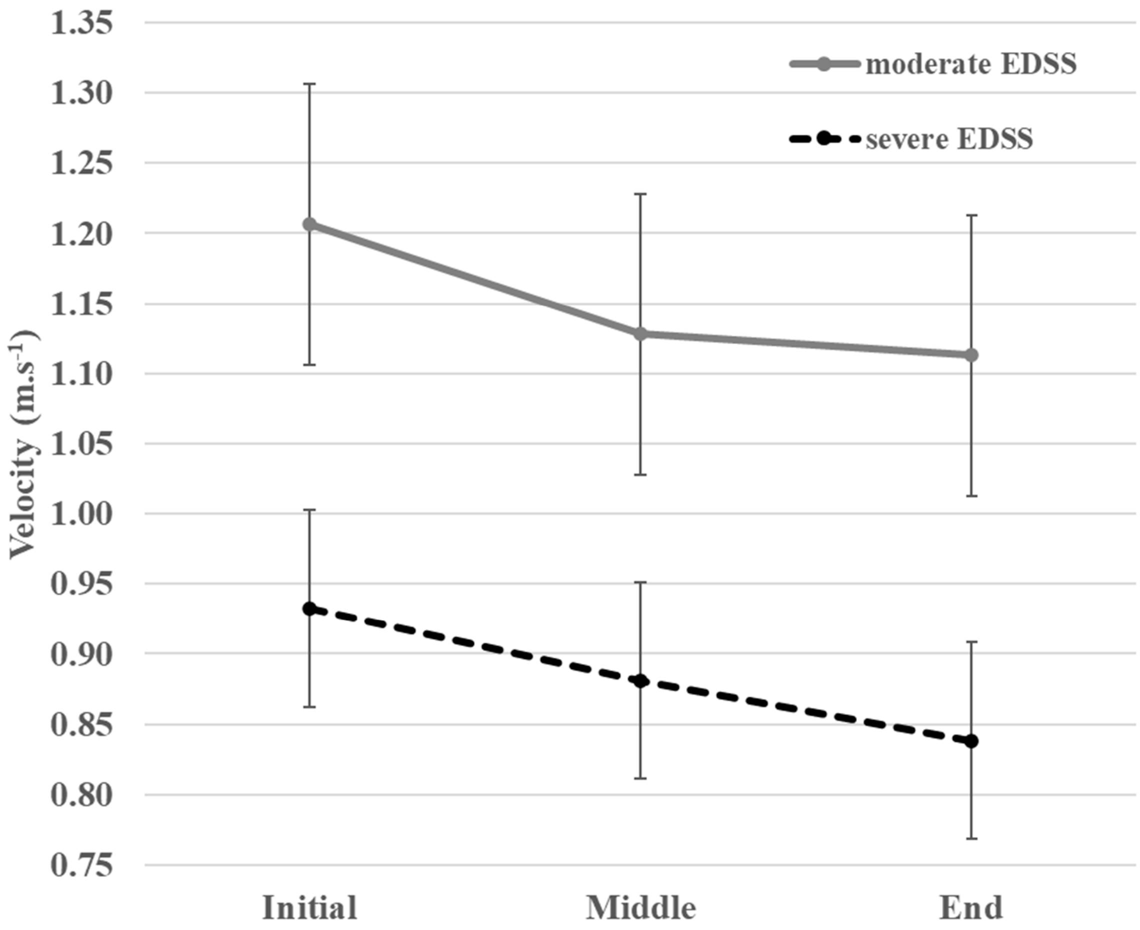

2

Moderate EDSS [4] (n=22)			
	6MWT interval		
**ST variables**	**Initial**	**Middle**	**End**
Velocity (m.s-1)	1.21 (0.24)	1.13 (0.24)	1.11 (0.24)
Cadence (step.min-1)	111.32 (10.52)	107.93 (9.48)	106.36 (9.88)
Stride length (m)	1.29 (0.20)	1.27 (0.21)	1.25 (0.21)
Stride width (m)	0.14 (0.04)	0.15 (0.04)	0.15 (0.04)
Double support time (% GC)	31 (4.18)	32 (4.21)	32 (4.62)

Severe EDSS [4.5-6.5] (n=23)			
	6MWT interval		
**ST variables**	**Initial**	**Middle**	**End**
Velocity (m.s-1)	0.93 (0.23)	0.88 (0.23)	0.84 (0.23)
Cadence (step.min-1)	100.06 (13.27)	95.11 (13.42)	91.97 (13.90)
Stride length (m)	1.11 (0.19)	1.06 (0.19)	1.02 (0.19)
Stride width (m)	0.14 (0.05)	0.15 (0.04)	0.15 (0.04)
Double support time (% GC)	34 (6.16)	36 (6.55)	37 (6.68)

Supplementary data SB: Spatio-temporal variables during each interval of 6MWT for PwMS with PP, RR and SP phenotype

1

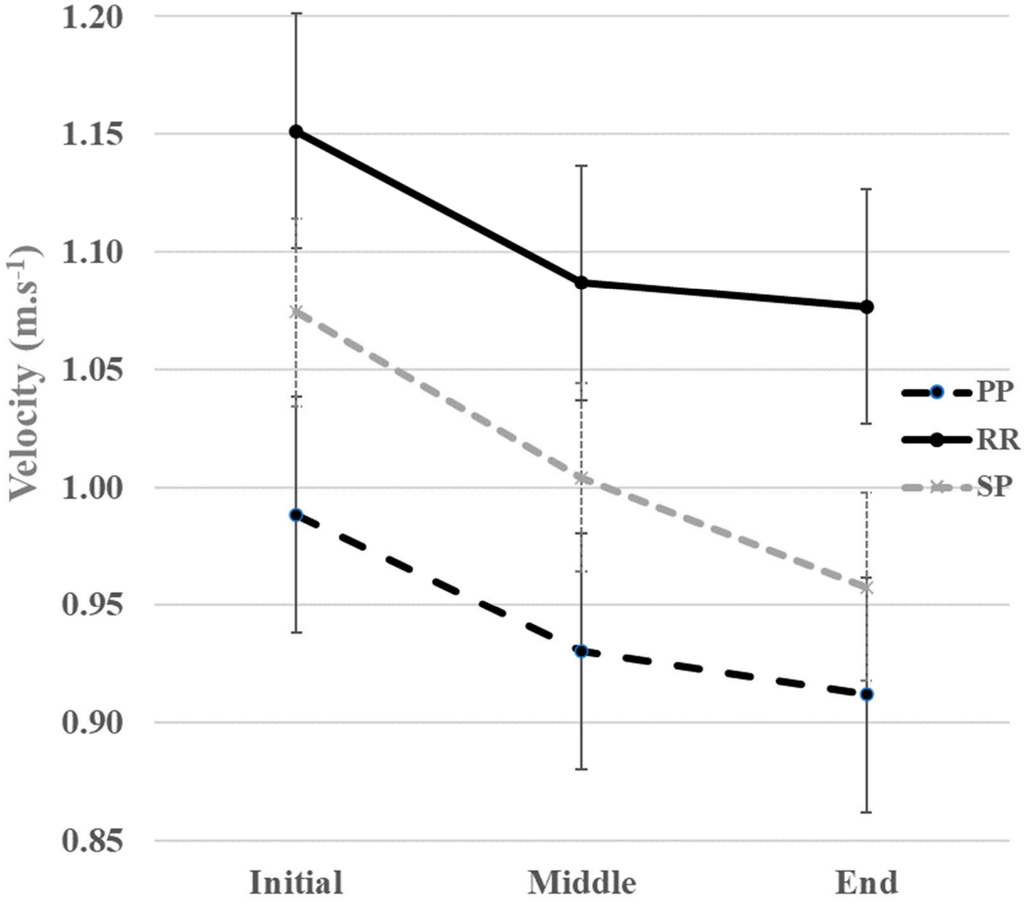

2

	6MWT interval								
	**PwMS with PP phenotype (n= 14)**			**PwMS with RR phenotype (n= 11)**			**PwMS with SP phenotype (n= 20)**		
**ST variables** [a]	Initial	Middle	End	Initial	Middle	End	Initial	Middle	End
Velocity ($m.s^{-1}$)	0.98 (0.31)	0.93 (0.32)	0.91 (0.31)	1.15 (0.22)	1.09 (0.25)	1.08 (0.20)	1.07 (0.26)	1.00 (0.25)	0.96 (0.27)
Cadence ($step.min^{-1}$)	101.98 (15.16)	97.66 (15.49)	96.61 (15.46)	109.63 (7.00)	105.78 (6.88)	104.30 (7.02)	105.83 (14.12)	101.55 (13.96)	98.06 (14.62)
Stride length (m)	1.14 (0.27)	1.12 (0.27)	1.11 (0.28)	1.25 (0.20)	1.22 (0.23)	1.21 (0.20)	1.21 (0.17)	1.17 (0.18)	1.14 (0.19)
Stride width (m)	0.14 (0.03)	0.15 (0.04)	0.15 (0.04)	0.12 (0.03)	0.13 (0.03)	0.14 (0.03)	0.14 (0.06)	0.15 (0.05)	0.15 (0.05)
Double support time (% GC)	35 (7.31)	36 (7.91)	37 (7.85)	32 (4.27)	33 (4.40)	34 (4.44)	31 (4.07)	32 (4.25)	32 (4.72)
